# Squaraine Nanodunes: Structure-Correlated Optical and Vibrational Anisotropy and Morphology-Enhanced Local Field Considerations

**DOI:** 10.1021/acsphotonics.5c01560

**Published:** 2025-10-14

**Authors:** Frank Balzer, Manuela Schiek

**Affiliations:** † Center for Surface- and Nanoanalytics (ZONA), 27266 Johannes Kepler University Linz, Altenberger Str. 69, 4040 Linz, Austria; ‡ Institute for Physical Chemistry (IPC) & Linz Institute for Organic Solar Cells (LIOS), Johannes Kepler University, Altenberger Str. 69, 4040 Linz, Austria

**Keywords:** squaraine thin films, Raman microscopy, polarization, Davydov splitting, field-enhancement

## Abstract

Squaraine thin films are emerging as functional optoelectronic elements because of their anisotropic optical properties in the visible to near-infrared spectral range, which include characteristic properties such as large Davydov splitting, hybrid Frenkel-charge transfer excitons, and giant circular dichroism. The prototypical squaraine 2,4-bis­[4-(*N,N*-diisobutylamino)-2,6-dihydroxyphenyl]­squaraine (SQIB) condenses into two different polymorphs (orthorhombic and monoclinic unit cells), both with distinct optical, electronic, and morphological properties. Polarized Raman microscopy spectra can distinguish between these polymorphs and indicate their crystallographic alignment, similar to polarized transmission spectroscopy, while atomic force microscopy precisely maps all topographical features. During crystallization, periodic nanodunes with cracks and protrusions along the local *c*-axis can form from the orthorhombic SQIB polymorph without any lithographic steps. The full dielectric tensor is known for this polymorph, and the components of the real part are strongly negative near the absorption bands. For metallic nanoparticles, it is known that a negative dielectric function can lead to localized surface plasmons and field confinement. In this study, we investigate which of the morphological features—nanodunes, cracks, or protrusions—have the potential to influence the excitonic properties in a similar enhancing fashion.

## Graphical abstract

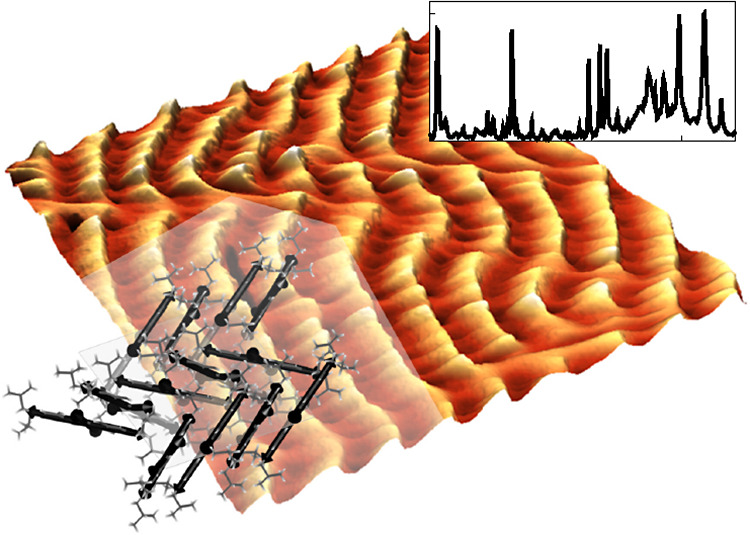

## Introduction

Organic semiconductors have become increasingly important in optoelectronics due to their tunable optical and electronic properties. Among these, squaraine dyes are of particular interest because of their exceptional optical characteristics, which are governed by strong excitonic effects.^1–5^ Squaraines offer high environmental and photostability and significant absorption in the visible and near-infrared spectral range, making them suitable for a variety of optoelectronic applications such as photovoltaic devices and photodetectors including chiroptical and biomedical sensing.^6–14^ Although commercial applications have not yet been realized due to comparatively low device efficiencies, squaraines are unique model compounds for characteristic aggregation, including color polymorphism,^15^ and structure-correlated excitonic coupling. These excitonic properties include Frenkel excitons with pronounced Davydov splitting, hybridization of Frenkel and charge transfer excitons, and excitonic circular dichroism boosted by charge transfer interactions.^16–23^ Frenkel excitons in general have also raised interest for the design of excitonic circuits and devices for quantum information science.^24,25^

A prominent squaraine dye is 2,4-bis­[4-(*N,N*-diisobutylamino)-2,6-dihydroxyphenyl]­squaraine (SQIB), Figure 1a, which crystallizes in two different polymorphs (monoclinic *P*2_1_/*c*, CCDC code 1567209, and orthorhombic *Pbcn*, CCDC code 1567104), whose distinct optical properties are well characterized.^16,26^ Both polymorphs show pronounced signatures of a Davydov splitting, with the complete spectrum of the orthorhombic polymorph being red-shifted, while the monoclinic polymorph shows a completely blue-shifted spectrum compared to the sharp absorption maximum of the monomer in solution.^27^ For the orthorhombic polymorph, even the full diagonal dielectric tensor is known, which is composed of three orthogonally polarized components that follow the directions of the unit cell axes.^28^ This implies a multiple Davydov splitting including a dark state:^23^ The two apparent Davydov bands in (polarized) absorbance spectra under normal incidence consist of an upper Davydov component (UDC) and an effective lower Davydov component (LDC), which is actually a superposition of the two spectrally close middle and lower tensor components. In addition, the dielectric tensor quantifies intense interactions with light, which manifest in strongly negative real parts of the dielectric function near the absorption bands. Such a negative real part of the dielectric function is a prerequisite to support plasmon modes. Metallic nanoparticles are typical plasmonic materials, and their negative permittivity is a consequence of the response of free Drude-type charge carriers to an external electric field.^29–32^ Excitonic organic materials exhibit negative permittivity due to a strong absorption band resulting from localized Frenkel or Frenkel-charge transfer hybrid excitons.^22,28^ Although the reason for the negative permittivity is different, nanostructured excitonic organic materials may be an alternative to achieve optical field confinement and enhancement.^33,34^

**Figure 1:**
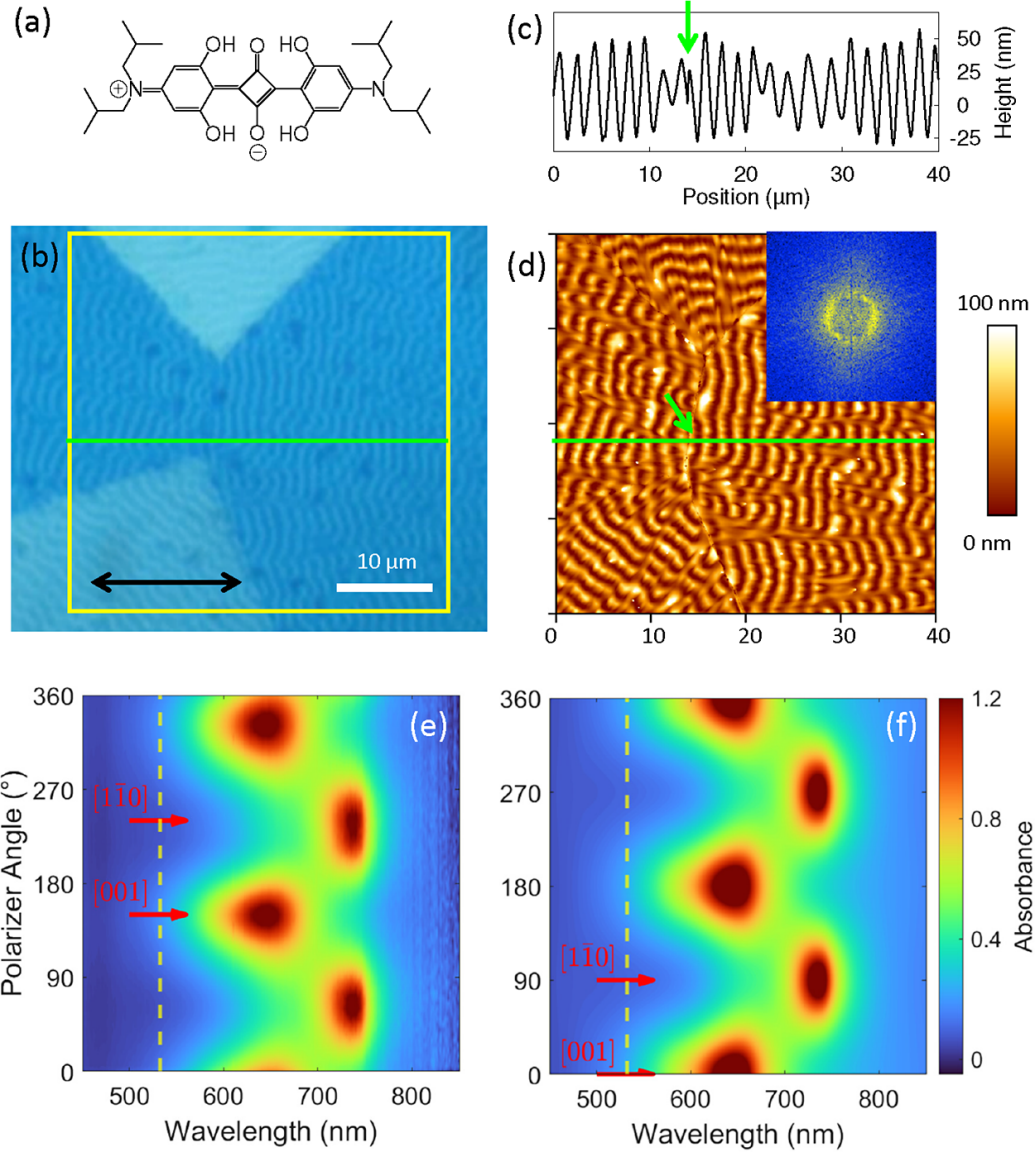
(a) Schematic molecular structure of SQIB in a zwitterionic resonance formula. (b) Polarized white-light optical transmission microscope image of orthorhombic SQIB nanodunes on glass, identifying four different SQIB rotational domains. The linear polarization of the light is along the black horizontal arrow. An AFM image (d) of the same area (yellow square) together with a cross-section along the green horizontal line (c) provides the morphology of the sample. The two-dimensional Fourier-transform inset in (d) demonstrates the periodic nature of the dune-like structures with a period of ≃1.9 μm. The green arrows in (c) and (d) mark a domain boundary. Measured (e) and calculated^40^ (f) normal incidence absorbance spectra for a 50 nm thick film of the orthorhombic SQIB polymorph with its (1 1 0) plane being parallel to the glass substrate. Note that only differences in polarizer angle are relevant, absolute values depend on the local [0 0 1] direction. The yellow dashed vertical lines mark 532 nm, the wavelength of the Raman excitation.

The choice of substrate, deposition conditions and annealing temperature determines which SQIB polymorph is formed, allowing controlled tailoring of the material properties.^35^ However, the preferred orientation of the individual polymorphs is determined by the intermolecular interactions, so that the stacking direction is parallel to the substrate. The orthorhombic polymorph forms extended rotational domains (platelets) with the (1 1 0) plane parallel to the substrate, whereas the monoclinic polymorph crystallizes into smaller domains in the submicrometer range with the (0 1 1) plane toward the substrate.^16^ The orthorhombic platelets are not necessarily single-crystalline, but can sometimes even spontaneously form periodic surface wrinkles (nanodunes) with dimensions comparable to those of visible to near-infrared light, particularly relevant for photonic applications. In organic semiconductor thin films, periodic surface undulations can spontaneously arise when the material is annealed above its glass transition temperature. This phenomenon results from a delicate interplay between crystal growth and surface mass transport, leading to self-organized structures with well-defined periodicity.^36^ The wavelength of these undulations has been found to depend on processing parameters, typically ranging from 800 to 2400 nm. Such bottom-up, self-assembly driven pattern formation is particularly attractive due to its inherent scalability and cost-effectiveness.^37^ Therefore, self-assembled periodic structures in organic semiconductors have been proposed for applications in light trapping in solar cells, enhanced light extraction in emissive devices, and organic microstructured lasers.

Here, we investigate the added value of periodic nanodunes, including cracks and protrusions along the local *c*-axis in thin films of orthorhombic crystalline textured SQIB focusing on their impact on light absorption, excitonic coupling, and polarization-dependent spectral characteristics. Polarized Raman microscopy together with polarized UV–vis transmission spectroscopy serves to distinguish between the two polymorphs and to indicate their crystallographic in-plane alignment, while AFM is used to precisely map all topographical patterns. Given the known anisotropic dielectric function with strongly negative real parts near the absorption bands of the orthorhombic SQIB polymorph, we also examine the potential for local field enhancement effects on nanoscale surface features.

## Results and Discussion

### Polarized Optical Properties and Nanodune Morphology

The morphology and optical properties of SQIB thin films were investigated using a combination of microscopy techniques and spectroscopic tools to understand how their self-organized periodic features influence light-matter interactions. Figure 1 provides an overview of a spin-casted and thermally annealed thin film from its orthorhombic polymorph formed on glass, resulting in the formation of platelets, some of them exhibiting periodic height modulations, i.e. nanodunes. Figure 1b presents a linearly polarized optical transmission microscope image of such a nanodune region, revealing four distinct domains. These domains are characterized by different shades of blue due to linear dichroism, indicating variations in in-plane crystalline orientation. The coupling between symmetry equivalent but translationally inequivalent molecules in the primitive unit cell (number of molecules *Z* = 4) results in a Davydov splitting, from which the projection onto the (1 1 0) plane is seen in normal incidence transmission spectroscopy. In this projection two absorbance bands appear that are shifted in their maximum polarized absorbance by 90° azimuthal rotation of the linear polarizer.^16^ Transitions to the upper Davydov split level (upper Davydov component, UDC) are located around 640 nm, transitions to the lower Davydov split level (lower Davydov component, LDC) around 740 nm. The UDC direction is parallel to the SQIB *c*-axis, the LDC direction is parallel to the SQIB [1 1̅ 0] direction, which is the direction of the projected *a*- and *b*-axes. Thus, the measurable effective LDC consists of a superposition of the two spectrally close tensor components along the *a*- and *b*-axes.^28^ In Figure 1, the measured (e) and calculated (f)^38–40^ polarized absorbance spectra are shown as a function of the polarizer angle at azimuthal rotation and wavelength with a view normal to the (1 1 0) plane. The experimentally determined 90° difference in the polarization angle for maximum absorbance between the two distinct peaks (UDC and effective LDC) is fully reproduced by the calculation based on the experimentally determined dielectric tensor.^28^ Other orientations of the sample would change the spectral signatures, which are composed of the three dielectric tensor elements; see the three examples in the Supporting Information in Figure S4 together with the dielectric tensor components, in comparison to the (1 1 0) orientation and the effective dielectric function from eq 6 in Figure S5a, and to the (1 1 1) orientation in Figure S5b.

The AFM image in Figure 1d, corresponding to the yellow square in Figure 1b, offers a detailed topographical view of the SQIB nanodunes. The periodic nature of these structures is evident, the Fourier transform inset further confirming its periodicity of approximately 1.9 μm.^41^ Figure 1c provides a cross-sectional height profile along the green horizontal line in Figure 1b,d, with a domain boundary marked by a green arrow. This profile reveals the undulating nature of the nanodunes, with heights varying between 25 and 75 nm.

The formation process of the nanodune structures is captured in a time series of polarized reflection microscopy images shown in Figure 2. These images were taken from a movie, which is available in the Supporting Information. Note that whereas in transmission microscopy the domains look blue, in reflection they have a brown-golden appearance. These time series images illustrate the crystallization of the orthorhombic SQIB polymorph on a glass substrate placed on a preheated sample holder at 180 °C. The SQIB layer was previously deposited on the substrate by spin-coating at room temperature from a chloroform solution, whereby an amorphous layer was initially formed. After a few seconds on the hot plate, when the top surface reaches a critical temperature, the crystallization starts and is easily observed by the development of birefringent and dichroic domains. Domain growth proceeds radially from nucleation sites, where the monoclinic polymorph is often found, Figure 3a. The presence of nanodunes is seen as an intensity variation. The nanodunes evolve with the growth process and their front is often oriented perpendicular to the radial growth direction. Domain growth stops when a growth front encounters another one, forming a domain boundary. The conditions for the formation of nanodunes are not entirely known, especially as they only form in some of the platelets on the sample. Their formation seems to depend on the kinetics of crystallization and interactions with the substrate surface. In any case, the period and height of the nanodunes depend on the spin-coating parameters; see Figure S3 in the Supporting Information. All other parameters being equal, on average larger platelet domains are formed with increasing acceleration and terminal velocity of the spin-coater.^28^ As the spin speed increases, the height and period of the nanodunes decrease. Their average maximum height^41^ *R*_tm_ varies from (70 ± 10) nm to (50 ± 10) nm, while the period decreases from (2.2 ± 0.2) μm to (1.3 ± 0.2) μm.

**Figure 2:**
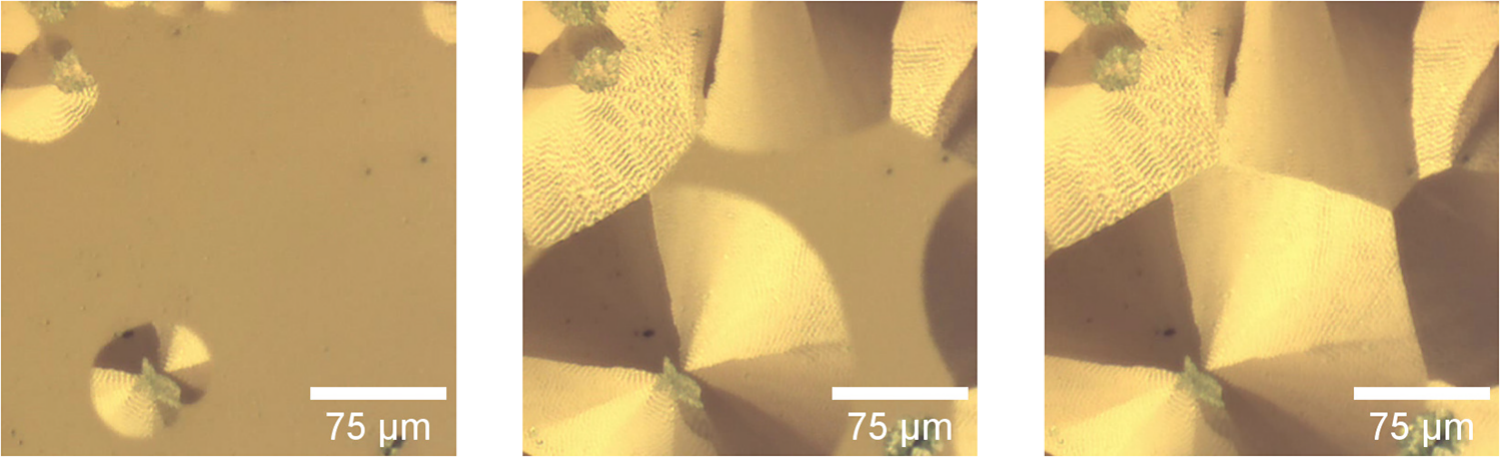
Time series of white-light polarized optical reflection microscope images during the crystallization (*t* = 3.7, 4.2, and 4.5 s from left to right) of the orthorhombic SQIB polymorph on glass, placed on a hot-plate with a temperature of 180 °C. Nanodunes form during crystallization, the whole crystallization process lasts only a few seconds. The entire movie is available in the Supporting Information section.

**Figure 3:**
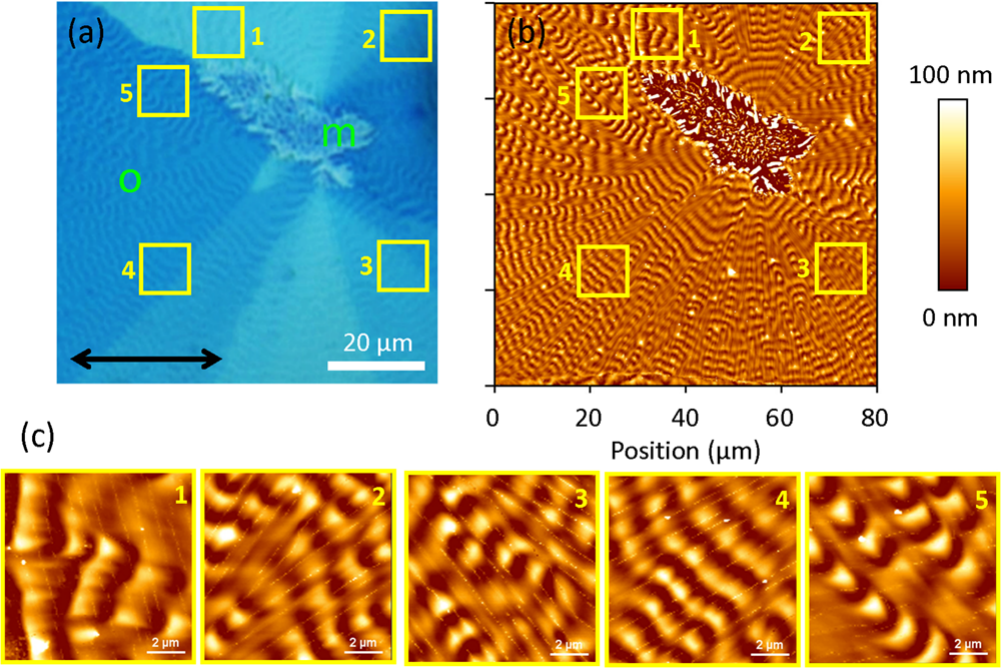
(a) Polarized white-light transmission microscope image and (b) the corresponding AFM image of the two common SQIB polymorphs, orthorhombic “o” and monoclinic “m”. The polarizer direction is along the horizontal (black arrow). Domain boundaries in platelets from the orthorhombic polymorph are visible as different shades of blue in (a). The directions of cracks and thin line-like protrusions in the orthorhombic layer are magnified in (c) for five different regions. Often these lines are perpendicular to the dune fronts, but not always, see also Figure S1.

Figure 3 presents more details on the polymorphic nature of spin-casted and subsequently annealed SQIB thin films on glass and about the in-plane alignment of the domains of the orthorhombic polymorph. The polarized transmission microscope image, Figure 3a, and the AFM image, Figure 3b, reveal the presence of the two distinct SQIB polymorphs. The orthorhombic polymorph is characterized by large platelets (tens of micrometers in size) with clear domain boundaries, suggesting the presence of different in-plane crystalline orientations within the same polymorph. The monoclinic polymorph, in contrast, forms smaller crystallites with submicrometer dimensions. Figure 3c magnifies regions of the orthorhombic layer. Thin parallel lines of either cracks or protrusions (width typically a few 10 nm, height about 15 nm), Figure S2, often run perpendicular to the dune structures, although this orientation is not completely universal. More detailed images can be found in Figure S1 to help understand the molecular arrangement within the platelets. It has already been shown that these lines follow the polarization direction of the UDC in the UV–vis transmission spectra. Thus, they run along the orthorhombic SQIB *c*-axis, which is aligned parallel to the substrate.^42^ The orientation of these lines serves as a simple marker for in-plane orientation of the orthorhombic SQIB platelets.

### Polarized Raman Microscopy Mapping

Raman microscopy is widely employed to elucidate molecular orientations in organic thin films and alignment of 2D materials.^43–45^ The observed Raman scattering intensity *I*(ϕ) as a function of azimuthal sample rotation ϕ is determined by the Raman tensor associated with a given normal mode and is proportional to

$$I(\phi )\propto \sum_{{\vec{e}}_{s}}|{\vec{e}}_{0}^{T}\cdot R(\phi )\cdot {\vec{e}}_{s}^{*}{|}^{2}$$ 1

where ${\vec{e}}_{0}$ and ${\vec{e}}_{s}$ are the polarization directions of the incident and scattered electric fields, respectively, and *R* = ∂α/∂*Q* is the Raman tensor for the normal mode with the polarizability tensor α. The symmetry of the normal mode determines the structure of the tensor *R*, which in turn defines the selection rules and polarization dependence of the Raman response. The orientation of an anisotropic sample domain ϕ relative to the excitation polarization adds a rotation-dependent modulation of the Raman intensity. Note that in contrast to the optical measurements, where alone the excitonic transitions of a crystalline sample are seen, Raman measurements show both molecular and crystalline modes of a crystalline sample.

The orthorhombic SQIB polymorph adopts a *Pbcn* space group with four molecules per unit cell (*Z* = 4) and half a molecule in the asymmetric unit (*Z*′ = 0.5). This means that each molecule resides on an inversion center and therefore exhibits *C*_*i*_ point symmetry. At the molecular level, the presence of an inversion center restricts the vibrational modes to either the *gerade* (*A*_*g*_) or *ungerade* (*A*_*u*_) irreducible representations, with only the *A*_*g*_ modes being Raman active. A SQIB molecule consists of 84 atoms that allow 3*N* – 6 = 246 vibrational modes, of which 123 are Raman-active *A*_*g*_ modes. These molecular *A*_*g*_ modes can be polarized along different directions relative to, e.g., the long molecular axis depending on the orientation of the polarizability derivative, i.e. how the vibration occurs relative to the long molecular axis. For the space group *Pbcn*, the crystal point group adopts a *D*_2*h*_ symmetry. As a consequence, each molecular *A*_*g*_ mode gives rise to four Raman-active vibrational modes in the crystal (internal lattice vibrational modes), transforming as the irreducible representations *A*_*g*_, *B*_1*g*_, *B*_2*g*_, and *B*_3*g*_. Each of the *B*_*ig*_ modes is associated with one of the crystallographic axes. The monoclinic SQIB polymorph adopts a *P*2_1_/*c* space group, belonging to the point group *C*_2*h*_, with *Z* = 2 and *Z′* = 0.5. Again, the *C*_*i*_ point symmetry restricts the Raman active vibrational mode to the *A*_*g*_ irreducible representations on the molecular level. In the crystal each normal mode of *A*_*g*_ symmetry transforms into one *A*_*g*_ and one *B*_*g*_ Raman-active mode. Explicit forms of the Raman tensors for the crystalline modes of the two SQIB polymorphs are given in the Supporting Information in eqs S1 and S2.

In polarized Raman spectroscopy, the *A*_*g*_ modes are basically observed in parallel polarization configuration with respect to incident and scattered light, while the *B*_*ig*_ modes appear only in cross-polarized configurations. Note that this only applies completely if the respective molecular or crystallographic axes are aligned with the excitation polarization. When the sample is rotated, the Raman tensor transforms, resulting in off-diagonal contributions and apparent cross-polarization components. See a comparison of measurements on two rotational platelet domains with different analyzer configurations in the Supporting Information, Figure S6. Vibrational Davydov splitting is on the order of 10 to 15 cm^–1^ and can be evident in the splitting of each molecular *A*_*g*_ mode in the crystalline environment: There are four Raman modes (*A*_*g*_ + 3*B*_*ig*_) for the orthorhombic polymorph and two Raman modes (*A*_*g*_ + *B*_*g*_) for the monoclinic polymorph, each with a characteristic polarization. To resolve and assign all Davydov components experimentally, both parallel and cross-polarized configurations must be measured and sample rotation must be accounted for.^46–48^

Our Raman measurements are conducted with linearly polarized excitation but without additional polarization analyzer, which allows to see both the symmetric (*A*_*g*_) and the antisymmetric (*B*_*g*_) Raman modes. However, polarization-specific selection rules are lost, and the ability to distinguish between Davydov components is significantly reduced. Therefore, we refrain from a discussion of vibrational Davydov splitting. However, also with linearly polarized excitation, Raman intensity mapping can exhibit a clear polarization dependence in crystalline domains. This is because the Raman tensor remains anisotropic, and its projection onto the excitation polarization depends on the orientation of the molecular vibrations within the crystal lattice. Consequently, even molecular *A*_*g*_ modes derived from centrosymmetric molecules can still show directional intensity variations due to the relative alignment of their polarizability changes with respect to the excitation polarization. In the particular case of orthorhombic platelets with (110) orientation, the sum of the spatial projections of the long molecular axes is predominantly aligned along the *c*-axis, Figure S1a. Raman molecular modes whose polarizability derivative lies along the SQIB long molecular axis are expected to exhibit greater Raman intensity for a sample orientation where the incident light is linearly polarized along the crystallographic *c*-axis.

Furthermore, the molecular *A*_*g*_ modes are not invariant properties of the isolated molecule but sensitive to the crystalline environment reflecting their local surroundings. Thus, they may exhibit slight but characteristic spectral shifts and intensity redistributions, which allow to distinguish between different polymorphs. The frequency shifts are expected to be on the order of a few cm^–1^.^49^ Raman modes with frequencies below 200 cm^–1^ are often more sensitive for distinguishing between different polymorphs. Such low frequency external crystal lattice vibrational modes (phonons) arise from collective motion of entire molecules.

Raman spectra of squaraines have already been investigated in the literature.^50,51^ Coffey et al.^52^ for example studied blends of an anilino squaraine (substituted with *n*-hexyl groups instead of *iso*-butyl found in SQIB) and the common fullerene PCBM ([6,6]-phenyl-C_61_-butyric acid methyl ester). In their analysis, characteristic Raman signals from PCBM served as spectral markers to identify and map the fullerene-rich domains. Raman spectra from aggregated regions of the *n*-hexyl anilino squaraine (nHSQ) closely resemble those of SQIB platelets, Figure 4, suggesting that the spectra are dominated by molecular Raman modes arising from the anilino squaraine backbone. The nHSQ crystallizes in a triclinic *P*-1 space group with *Z* = 1 and *Z*′ = 0.5.^53^ Both the space group and the nHSQ molecule adopt point group symmetry *C*_*i*_ that generates only molecular and crystalline *A*_*g*_ modes with comparable frequencies.

**Figure 4:**
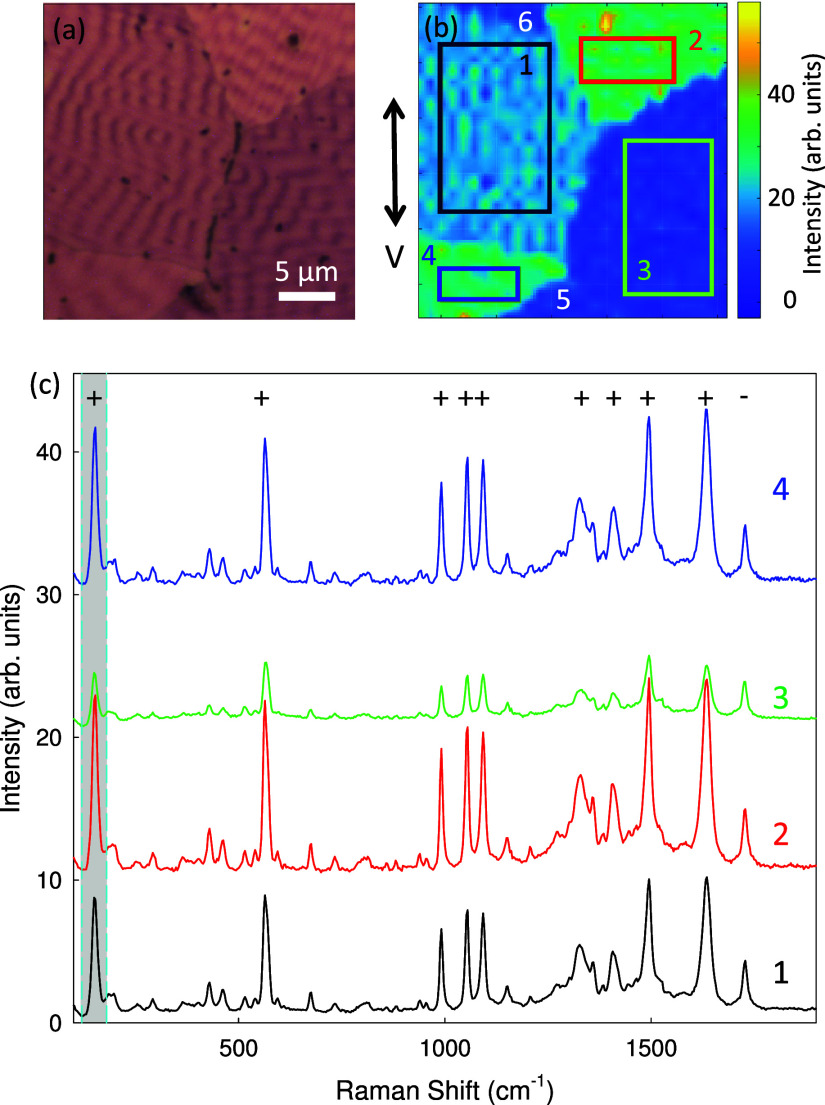
(a) Optical reflection microscopy image of a sample region. (b) Vertically (V)-polarized Raman map acquired with λ_exc_ = 532 nm (laser power 0.5 mW, 100× microscope objective), showing the integrated intensity of the Raman band between 120 to 180 cm^–1^ (gray-shaded area in c). Six distinct domains are visible in both images. Colored rectangles highlight four domains from which the averaged Raman spectra in (c) were obtained. Polarization dependence is indicated only for the strong Raman bands, with ‘–’/‘+’ denoting weak/strong dependence. Spectra are vertically offset for clarity.

Figure 4 explores the polarized Raman characteristics of the orthorhombic SQIB domains comprising nanodunes. As before, the domains differ in their in-plane orientation, whereas the out-of-plane orientation is fixed during the growth crystallization process to the (1 1 0) plane. The optical reflection microscopy image, Figure 4a, corresponds to the area where a vertically polarized Raman map was obtained (vertically polarized excitation, detection without polarization analyzer), Figure 4b. The Raman transitions used mainly in the present study are the one at 1730 cm^–1^, presumably the C=O carbonyl stretch vibration,^54^ the terminal C=C stretch vibration at 1620 cm^–1^, and the low energy (<200 cm^–1^) external crystal lattice vibrational modes (phonons) and ring-torsion modes. Based on the intensity of the first strong Raman band (120 to 180 cm^–1^), mapping clearly distinguishes six domains, of which four are marked. The spatially averaged Raman spectra for these domains, Figure 4c, reveal significant differences: The absolute and relative intensities of the peaks vary across domains, particularly noticeable for the peaks at 130 and 1620 cm^–1^ compared to, for example, the one at 1730 cm^–1^, which remains almost constant in intensity. Intense peaks with a strong polarization dependence are marked with ‘+’, while ‘–’ indicates a weak polarization dependence.

The overall Raman intensity is high when the polarization is parallel to the local crack direction, i.e. the excitation polarization is along [0 0 1], where the maximum amount of light from the UDC is absorbed, Figure 1e. The reason is the orientation of the long molecular axis of SQIB on average along the *c*-axis for the orthorhombic polymorph, Figures S1a and 5. This enables straightforward orientation mapping for Raman modes polarized along the molecular backbone, such as the terminal chain stretching vibration C=C at 1620 cm^–1^. In a crystalline textured platelet film, the 1620 cm^–1^ band has a maximum intensity when the linear polarization of the excitation light is parallel to the crystallographic *c*-axis, with an expected dependence on the azimuthal rotation angle ϕ. In contrast, the carbonyl stretching vibration (C=O) at 1730 cm^–1^ exhibits only a weak polarization modulation.

**Figure 5:**
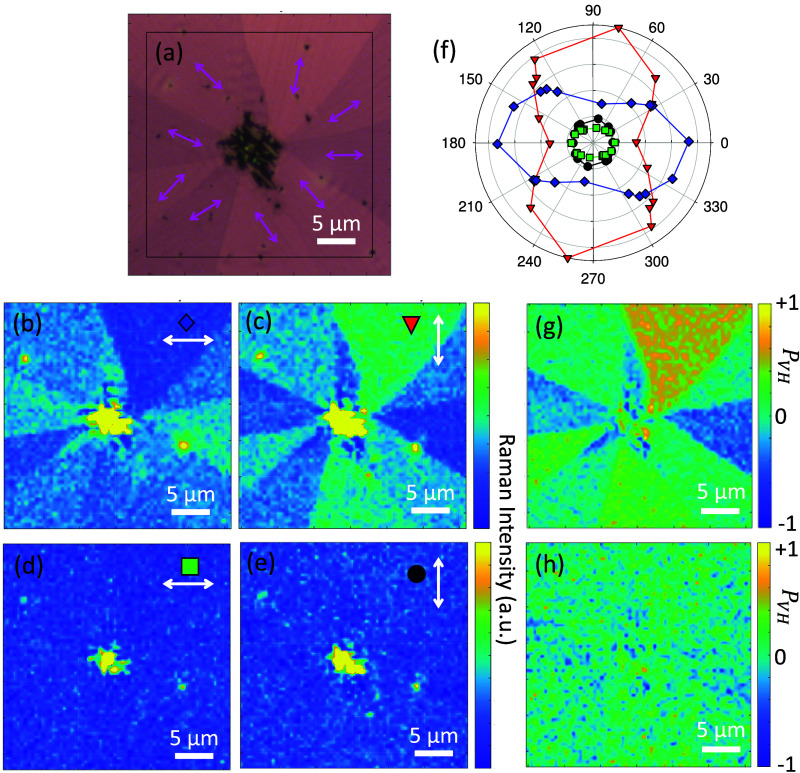
Polarization analysis of two Raman bands. The pink arrows in the optical microscope image (a) mark the directions of the cracks/protrusions within rotational domains (orthorhombic polymorph), thus the local *c*-axis. In the center crystallites of the monoclinic polymorph are situated. (b–e) Corresponding polarized Raman maps of the band at 1620 and 1730 cm^–1^. The polarization direction of the excitation laser is denoted by white arrows. The Raman intensity as a function of the direction of the local *c*-axis, symbols in (f), demonstrates the isotropic behavior of the 1730 cm^–1^ band (green squares and black circles), whereas the 1620 cm^–1^ band (blue rhombs and red triangles) results in maximum intensity when the excitation polarization is parallel to the local *c*-axis, i.e. parallel to the average long molecule axis, Figure S1a. The solid lines serve to guide the eye. The polarization ratio *P*_VH_ for the Raman band at 1620 cm^–1^ (g) is sensitive to the rotational domains, whereas the one at 1730 cm^–1^ is more isotropic (h). The laser power of the excitation laser was 0.05 mW, the microscope objective magnification 100×.

Using the information on crystallographic orientation within the domains provided by the direction of the cracks, a simple polarization-dependent Raman analysis avoiding full rotational scans becomes accessible. It provides further insight into the rotational domain alignment within crystallized SQIB films by exploiting the sensitivity of certain Raman bands to domain alignment. In Figure 5, the polarization dependence of two selected Raman bands for the orthorhombic polymorph is deterred from maps with polarized excitation, analyzing the Raman intensity of rotational domains. The local *c*-axis, i.e. the averaged local long molecule direction, is estimated from the crack/protrusions direction of the corresponding optical microscope image, pink arrows in Figure 5a. The 1620 cm^–1^ band has an anisotropic polarization response, Figure 5b,c, while the 1730 cm^–1^ band is isotropic, Figure 5d,e. Figure 5b,d are obtained for horizontally polarized laser excitation, 5c,e for vertically polarized excitation. Using this optical/morphological orientation identification, the angular dependence of the Raman intensity is plotted in Figure 5f. The 1620 cm^–1^ band shows maximum intensity for the local *c*-axis being horizontally for horizontally polarized excitation (blue diamonds), or vertically for vertically polarized excitation (red triangles), respectively, i.e. when the local *c*-axis orientation is aligned with the direction of the linear polarization. The Raman intensity is never zero as expected from the calculated overall angular pattern of the molecule orientation along the local crystallographic *c*-axis, Figure S1a, based on the projected average distribution of the long molecular axis of the four inequivalent molecules within the orthorhombic unit cell.

Another quantity to visualize the polarization dependence on domain orientation is the intensity ratio of the individual Raman peaks for horizontally (H) and vertically (V) polarized excitation, *I*_H_ and *I*_V_, respectively^43^

$$P_{\mathrm{VH}}=\frac{I_{V}-I_{H}}{I_{V}+I_{H}}$$ 2

Polarization ratio (*P*_VH_) maps for different Raman bands also reveal the sensitivity of certain vibrational modes to crystal orientation: The 1620 cm^–1^ band, Figure 5g reproduces the strong polarization dependence, with a clear contrast between different rotational domains. In contrast, the 1730 cm^–1^ band, Figure 5h, again shows little polarization dependence, indicating a more isotropic vibration or one that is less sensitive to crystal orientation.

In addition to the analysis of molecule orientations, Raman spectroscopic analysis can also provide insights into the differences between polymorphs. An example from the literature is copper phthalocyanine, where the bands near 1530 and 680 cm^–1^ shift by several wavenumbers between the α- and β-crystal forms,^55^ and other small molecular polymorphs such as pentacene and rubrene,^43,56,57^ providing an unambiguous Raman fingerprint for phase identification. Raman and resonance-Raman peak-shifts were also used to differentiate H- from J-aggregates of organic dyes.^58–60^ Figure 6a shows Raman spectra, where the reflection microscope image (b) from the same area demonstrates that both polymorphs are present. In (c), a Raman map of the 1620 cm^–1^ band for this region is shown, which clearly distinguishes between the orthorhombic and monoclinic regions, colored blue and yellow-green, respectively. Figure 6a compares the averaged and normalized to 130 cm^–1^ Raman spectra for orthorhombic (black line) and monoclinic (red line) polymorphs. Although the overall spectral features are similar, there are notable differences: First, the relative intensities of certain Raman bands vary between polymorphs. Some peaks are more pronounced in one polymorph compared to the other, which is illustrated by mapping the intensity ratio of the Raman bands at around 1620 and at 130 cm^–1^ in Figure 6d. The peak ratio is typically less than one for the monoclinic polymorph and equal to or even larger than one for the orthorhombic polymorph. Second, some peaks also show slight red or blue shifts by ≃5 to 10 cm^–1^ between the two polymorphs, indicating differences in molecular packing and intermolecular interactions. These peaks are marked by asterisks in Figure 6a. The exact peak position for the band around 1620 cm^–1^ is mapped in Figure 6e, consistent with the polymorph discrimination by relative peak intensities. Third, some Raman peaks are split by 5 to 10 cm^–1^ for the monoclinic polymorph compared to the orthorhombic one. In Figure 6a they are marked with the symbol ‘#’ and in Figure S7 a detailed view of these peaks is given. All these spectral differences therefore serve as a fingerprint for identifying and distinguishing between the two SQIB polymorphs.

**Figure 6:**
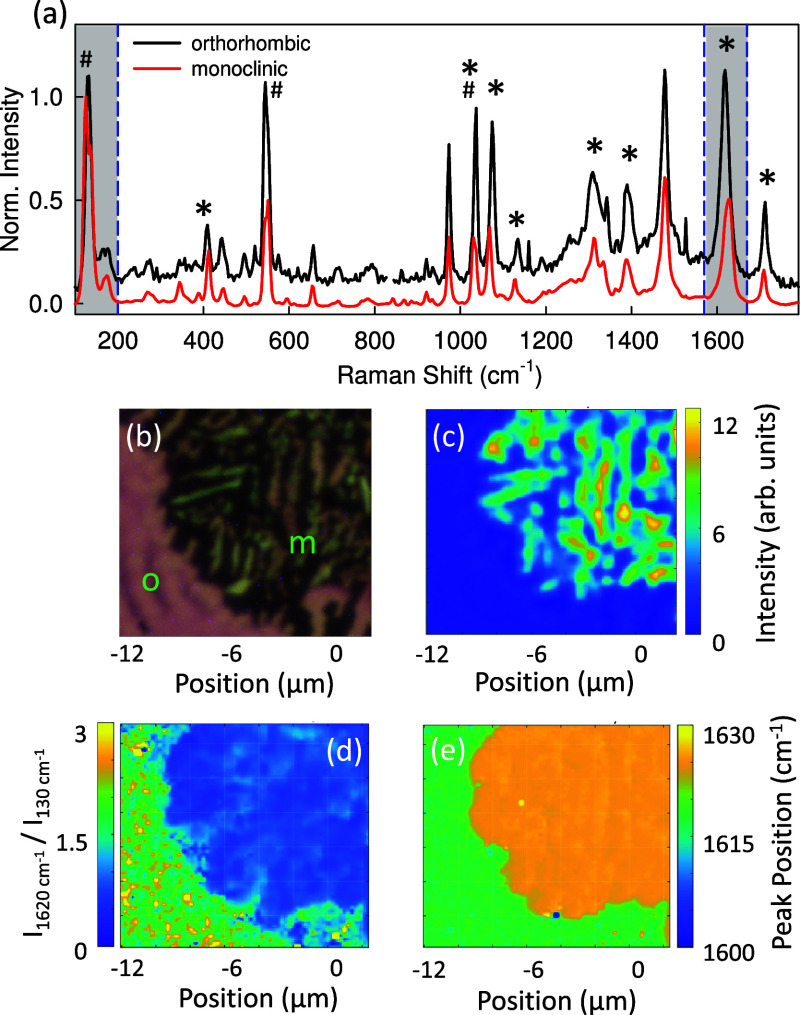
(a) Averaged Raman spectra of the orthorhombic (black) and monoclinic (red) polymorphs, obtained by summing spectra recorded with vertically and horizontally polarized excitation and normalized to the 130 cm^–1^ band. Gray-shaded regions indicate the spectral ranges used in (c–e). (b) White-light reflection image of the mapped area, and (c) Raman map of the integrated intensity of the 1620 cm^–1^ band (λ_exc_ = 532 nm, 0.05 mW laser power, 100× microscope objective). (d) Intensity ratio of the Raman bands at 1620 and at 130 cm^–1^ and (e) peak position of the 1620 cm^–1^ band. Differences between the two polymorphs are reflected in the relative band intensities (d) and peak shifts (e). In (a), ‘*’ marks Raman modes that differ in position by ≃ 5 to 10 cm^–1^, ‘#’ lines which are split. Spectra are vertically offset for clarity.

A close-up Raman map into a single orthorhombic SQIB domain with nanodunes, where individual dunes and valleys are resolved, is provided in the Supporting Information in Figure S8a. An intensity contrast is observed between the dunes and valleys, but no differences in the relative intensities or positions of the Raman peaks are noticeable, Figure S8b. The intensity variation is supposedly simply due to the variation in material thickness between the two areas with virtually no crystallographic realignment taking place. This means that the formation of nanodunes is a morphological feature. With this in mind, polarization-dependent Raman microscopy provides a powerful tool to map crystallographic alignment and resolve different domains in textured SQIB thin films.

### Morphology-Enhanced Local Field Analysis

In the next step, we consider the effects of nanodune morphology on the local excitonic properties of an orthorhombic SQIB thin film. We hypothesize that a tip effect possibly might cause field enhancement. Although the nanodunes are extended and elongated surface features, making their geometry more similar to one-dimensional cylinders rather than spherical nanoparticles, the fundamental mechanism of field enhancement remains governed by the dielectric function and boundary conditions. As such, qualitative trends in local field enhancement due to negative real parts of the dielectric function should persist across different geometries. In particular, both spheres and cylinders exhibit resonance conditions when their internal dielectric function satisfies specific relationships with the surrounding medium. For spherical particles with a dielectric function ε, this resonance – known as the Fröhlich condition – is reached when the real part of the complex dielectric function ε of the particle satisfies

Re(ε)=−2εm 3

where ε_m_ is the dielectric function of the embedding medium. For cylinders, the condition becomes Re­(ε) = – ε_m_, but the overall behavior remains similar. Thus, despite the deviation from perfect spherical symmetry, the field enhancement observed in the nanoparticle model provides a reasonable estimate for the enhancement behavior in the more realistic nanodune structures.

A typical tip radius of a nanodune perpendicular to the nanodune fronts is estimated from AFM images to be on the order of several hundred nanometers up to several micrometers. We can estimate the local electric field enhancement inside a nanosphere embedded in a dielectric medium using the quasi-static approximation, i.e., when the particle size is much smaller than the wavelength of the incident light:

$$\frac{E_{i}}{E_{0}}=\frac{3\varepsilon_{m}}{\varepsilon +2\varepsilon_{m}}$$ 4

This expression describes the amplitude of the internal electric field *E*_*i*_ relative to the incident field *E*_0_ for small spherical particles.^29^ The internal field enhancement reaches its maximum when the real part of the sphere dielectric function satisfies the Fröhlich condition Re­(ε) = – 2ε_m_. In vacuum, ε_m_ = 1, this simplifies to Re­(ε) = – 2. However, this requires that the imaginary part in the corresponding spectral range approaches zero, which is typically the case for metals, but not for the orthorhombic SQIB polymorph. In this case, the values for the dielectric function are about −2.0 + 1.8i at 572 nm along the UDC direction [0 0 1] and −2.0 + 3.6i at 698 nm along the effective LDC direction [1 1̅ 0], Figure S5a. The effective LDC is calculated from the dielectric tensor components along the *a* and *b* axes according to eq 6, see also Figure S5. This means that the expected field enhancement due to damping by the notable imaginary part of the dielectric function may be small, especially at the rather large tip radii of the nanodunes, which exceed the dimension of the given wavelength range by a factor of 10. For reasonably small J-aggregated dye-doped nanospheres it was shown, that such excitonic materials can achieve substantial optical field enhancement and subwavelength confinement when their resonant permittivity becomes sufficiently negative (Re­(ε) < −2) while the imaginary part remains moderate.^33^

Figure 7 shows the angular averaged mean-absolute-square internal electric field enhancement |*E*_*i*_/*E*_0_|^2^ at the inner nanosphere surface as a function of nanosphere radius and wavelength of the incident light,^29,61,62^ taking into account the dielectric function along the UDC direction in (a) and along the effective LDC direction in (b). In both cases, the maximum field enhancement does not occur at Re­(ε) = – 2 but at slightly different values of Re­(ε) and consequently smaller amplitudes of Im­(ε). This is the case when |ε + 2| reaches a minimum. Therefore, the spectral position of the maximum field enhancement is shifted to significantly shorter wavelengths. Unfortunately, the maximum field enhancement at about 625 nm is negligible for the effective LDC component, Figure 7b. Also, the maximum field enhancement at about 555 nm considering only the UDC dielectric tensor component is small, less than 5 for small nanospheres (radius smaller than 20 nm), Figure 7a. This trend can also be seen in the near-field distribution for nanospheres with a radius of 20 nm in Figure S9a,b. For comparison with nearly free-electron metal nanoparticles, the mean-absolute-square internal electric field enhancement |*E*_*i*_/*E*_0_|^2^ in a small Na nanosphere from eq 4 reaches almost 300 at λ = 378 nm in vacuum.^63–66^ Because of its low damping and high plasma frequency and without having considerable interband transitions in the visible range, sodium exhibits strong field enhancement, Figure S9c.

**Figure 7:**
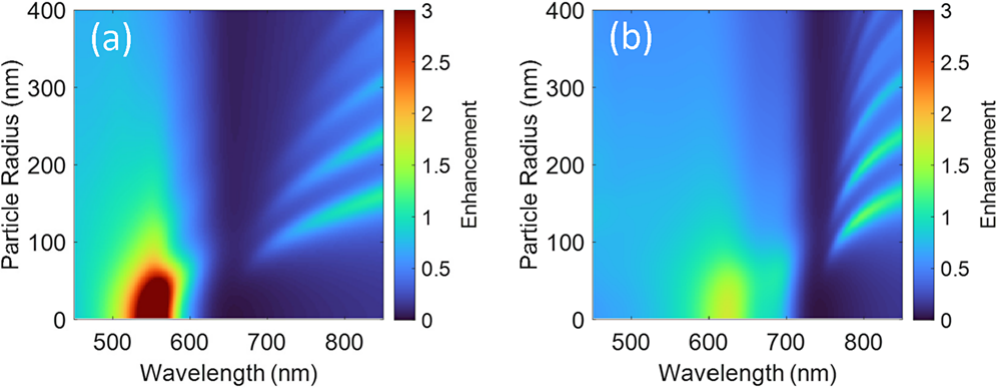
Mean-absolute-square internal electric field enhancement |*E*_*i*_/*E*_0_|^2^, averaged at the inner surface of SQIB nanospheres in vacuum and considering either the dielectric function along the [0 0 1] (UDC) direction (a) or along [1 1̅ 0], effective LDC, eq 6, (b).^28,29,61^

This means that a clearly negative real part of the dielectric function, which gives the SQIB platelets their golden shine in reflection, Figure 2, alone is not sufficient for an appreciable field amplification to support surface plasmon polaritons. Whereas the tops of the dunes are way too large for any considerable field enhancement because of their smooth curvature and micrometer-scale radius, the crystallites along the cracks and protrusions fall into a size regime where localized field enhancement effects may become relevant. The geometrical features such as the sharp protrusions reach lateral sizes below 100 nm and heights around 15 nm, which could allow for a significant field enhancement at or near the surface. This makes these features potentially useful for enhancing excitonic interactions locally, in analogy to the localized surface exciton-polariton modes discussed in the literature.^33^

## Conclusions

Although crystalline textured SQIB does not exhibit localized surface plasmon polaritons like metallic nanoparticles, it is distinguished by its exceptional excitonic properties, including a multiple Davydov splitting, which has recently attracted attention due to its fundamental theoretical importance.^23^ The complex morphology, here in particular nanodunes, and polymorphism make crystalline textured SQIB films attractive for investigation by various microscopic and spectroscopic techniques. To provide an estimate of possible field-enhancement effects, the “Ansatz” for spherical Mie particles embedded in vacuum (ε_m_ = 1) was chosen for its simplicity. While more elaborate approaches such as numerical solutions of the complete dune morphology or Mie-theory calculations for infinite cylinders are in principle available, this Ansatz provides a reliable model at far lower computational costs. As a result, field-enhancement effects can be ruled out for the nanodunes. We have discussed polarized Raman microscopy of both polymorphs in combination with a remarkable nanodune morphology and excitonic properties. Polarized Raman microscopy works similarly well as polarized spectro-microscopy to distinguish the two SQIB polymorphs and to determine the in-plane orientation of the orthorhombic platelets. Initial considerations on the Raman modes and their possible Davydov splitting provide a template for further conceptual studies on multiple Davydov splitting in vibrational spectra. SQIB is and remains a lovely looking model material for the design of excitonic molecular solids with a potential application in optoelectronics and photonics.

## Methods

Powder of 2,4-bis­[4-(*N,N*-diisobutylamino)-2,6-dihydroxyphenyl]­squaraine (SQIB), Figure 1a, was dissolved in amylene-stabilized chloroform at concentrations of 4 mg/mL to 6 mg/mL. Spin-coating with variable spinning parameters was carried out either in a glovebox under an inert nitrogen atmosphere or under ambient conditions in a flow box. The annealing of the samples was performed in an inert atmosphere (sample series in Figure S3) or under ambient conditions (time series samples in Figure 2 and Raman microscope samples in Figures 6, 5, and 4 on a hot plate). Inert or ambient conditions have a negligible influence on nanodune formation, while the spin-coating parameters control their dimensions, Figure S3. In all samples discussed here, the surface temperature of the preheated hot plate was always set at (180 ± 5) °C.

Atomic force microscopy (AFM) was conducted using a JPK NanoWizard in intermittent contact mode employing standard tips (BudgetSensors Tap300-G, Nanosensors NCH, and Nanosensors SSS-NCH cantilevers, all with a resonance frequency of about 300 kHz and a force constant of approximately 42 N/m). Image analysis was performed using the AFM manufacturer’s data processing software, or Gwyddion^41^ and ImageJ.^67^ Crystal structures are visualized with VESTA.^68^

Time-resolved optical microscopy was carried out in reflection geometry using an optical microscope equipped with a heatable sample holder. The temperature was monitored by a K-type thermocouple positioned directly adjacent to the sample. For the basic polarized optical characterization, a polarization microscope (Leitz DMRME) was used in combination with a fiber-optics miniature spectrometer (Ocean Optics Maya2000), coupled via a 200 μm diameter fiber to the camera port of the microscope. The sample was rotated in steps of 5° over 360° by a computer-controlled rotation stage (Thorlabs PRM1Z8). For each angle, a transmission spectrum was taken.

Raman microscope images were collected using a LabRAM Aramis VIS system, using a green laser with powers ranging from 0.05 mW to 0.5 mW at 532 nm and employing either a 50× or a 100× objective. Notably, bleaching and degradation of the films has been observed for laser powers of 0.5 mW and above. The linear polarization of the excitation laser can be rotated from “vertical” to “horizontal” using a waveplate. For detection of the Raman signal, an analyzer with vertical or horizontal linear polarization can be selected.^69^ The Raman spectra shown were all recorded without an analyzer. The polarization sensitivity of the spectrometer grating only produces a constant overall scaling of the detected signal, since the polarization of the Raman signals are determined by the polarization of the excitation according to the selection rules and the relative alignment of the respective mode. This means that the maxima/minima in the intensity profile of rotational domains remain unaffected.

Calculations of the field-enhancement use the theory described by Bohren and Huffman^29^ and were implemented with code by Mätzler and PyMieLab.^61,62^ The calculations are solely based on the dielectric function, previously determined by Mueller-Matrix ellipsometry.^28^ Polarized absorbance spectra are calculated using either a self-written transfer matrix code^70^ or code based on PyLlama.^40^

The dielectric tensor of the orthorhombic SQIB polymorph has been determined^28^ as

$$\varepsilon =(\begin{array}{ccc} \varepsilon_{\alpha } & 0 & 0\\ 0 & \varepsilon_{\beta } & 0\\ 0 & 0 & \varepsilon_{\gamma } \end{array})=(\begin{array}{ccc} N_{c}^{2} & 0 & 0\\ 0 & N_{a}^{2} & 0\\ 0 & 0 & N_{b}^{2} \end{array})$$ 5

where the indices *a*, *b*, and *c* denote the crystallographic axes, and the indices α, β, and γ order the components with respect to their excitonic transition energies from high to low, Figure S4. The lengths of the primitive unit cell axes for the orthorhombic polymorph are *a* = 15.0453 Å, *b* = 18.2202 Å, and *c* = 10.7973 Å. For the transfer matrix code, birefringence was included via effective refractive indices.^38^ These were obtained by rotating the impermeability tensor η = ε^–1^ in the laboratory frame, determining the eigenvalues λ_1,2_ of the relevant sub-block, and calculating $N_{{\mathrm{eff}}_{1,2}}=\frac{1}{\sqrt{\lambda_{1,2}}}$ .^71,72^ In the case where the (1 1 0) plane of SQIB is parallel to the thin film surface, η must be rotated by θ = 50.452° around the *x*-axis. As a result, one refractive index for light propagating along the *z*-axis remains *N*_*c*_, while the other becomes

$$N_{\mathrm{eff}}={\left(\frac{\cos^{2}\theta }{N_{a}^{2}}+\frac{\sin^{2}\theta }{N_{b}^{2}}\right)}^{-1/2}=\frac{N_{a}N_{b}}{\sqrt{N_{a}^{2}\sin^{2}\;\theta +N_{b}^{2}\cos^{2}\;\theta }}$$ 6

## Supplementary Material





## Notes

Figure S1, crystal structure of the orthorhombic SQIB polymorph, the (1 1 0) plane being parallel to the plane of the paper, together with an AFM image of rotational SQIB domains including cracks and protrusions; Figure S2, AFM images of protrusions; Figure S3, sample series of SQIB for different spin-casting parameters, where the annealing was performed in an inert atmosphere; Figure S4, calculated polarized absorbance spectra for low-index faces of the orthorhombic SQIB polymorph and the corresponding dielectric tensor components; Figure S5, same as Figure S4, but for the SQIB (1 1 0) and (1 1 1) faces; Figure S6, full polarization resolved Raman spectra from two domains; Figure S7, Raman spectra of the split lines for the two SQIB polymorphs; Figure S8, Raman maps for SQIB dunes; and Figure S9, electric field enhancement in SQIB nanospheres, compared to a Na nanoparticle (PDF)
Crystallization of an orthorhombic platelet film (AVI)

## §

University of Duisburg-Essen, Faculty of Engineering, Bismarckstr. 81, 47057 Duisburg, Germany
