# Supplementary material for: Squaraine Nanodunes: Structure-Correlated Optical and Vibrational Anisotropy and Morphology-Enhanced Local Field Considerations
Source: ACS Photonics. 2025 Oct 14;12(11):6103–13. doi: 10.1021/acsphotonics.5c01560 (PMC12636066; doi:10.1021/acsphotonics.5c01560)
Supplement: Supplementary file 1 [file ph5c01560_si_001.pdf]

Supporting Information: Squaraine Nanodunes: Structure-Correlated Optical and Vibrational Anisotropy and Morphology-Enhanced Local Field Considerations

Frank Balzer[*,†,¶] and Manuela Schiek[*,†,‡]

†Center for Surface- and Nanoanalytics (ZONA), Johannes Kepler University Linz, Altenberger Str. 69, 4040 Linz, Austria

‡Institute for Physical Chemistry (IPC) & Linz Institute for Organic Solar Cells (LIOS), Johannes Kepler University, Altenberger Str. 69, 4040 Linz, Austria

¶Current address: University of Duisburg-Essen, Faculty of Engineering, Bismarckstr. 81, 47057 Duisburg, Germany

E-mail: frank.balzer@uni-due.de; manuela.schiek@jku.at

Nanodunes, Cracks, Protrusions, and Crystallography

The SQIB nanodunes are formed by the orthorhombic SQIB polymorph, Figure S1(a), with the (1 1 0) plane being parallel to the substrate. Fine cracks and protrusions in the films are along the [0 0 1] direction, blue lines in Figure S1(b). Within different domains, these directions are all mutually parallel. One of those domain boundaries together with protrusions are shown in more detail in Figures S2(a) and (b). Often, the dunes are oriented perpendicular to the crack/protrusion directions. Note that for the orthorhombic polymorph the four possible directions of the long molecule axes, red arrows in Figure S1(a), do not average out, but lead to an average long molecule axis along the c-axis.

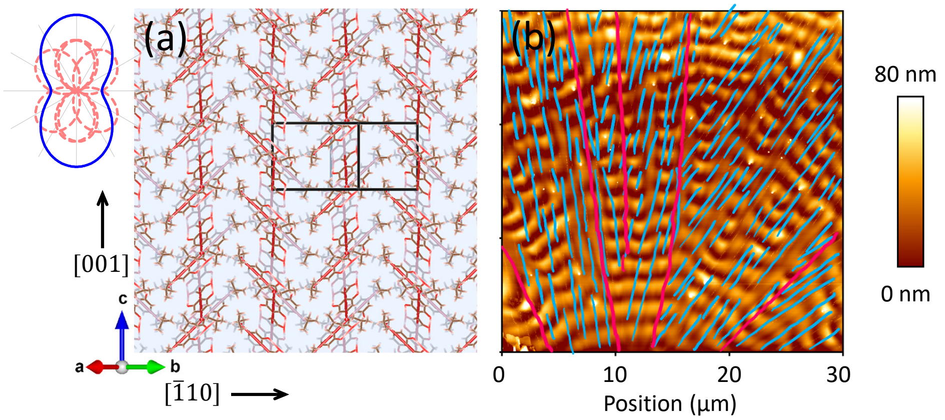

Figure S1: (a) Structure of the (1 1 0) surface of the orthorhombic SQIB polymorph.[1] The projected primitive unit cell is shown by black lines, the directions along the axes of the projected unit cell in the surface plane are indicated by black arrows. Red arrows mark the individual molecule's long axes. Assuming a $\cos^2 \phi$ excitation profile for each molecule along the long molecule axis (shown schematically by red dashed lobes), the incoherent angular average over molecular orientations yields the blue directional emission pattern. In (b), cracks/protrusions and domain boundaries of a typical sample are marked by blue and red lines, respectively, on an AFM image. Cracks and protrusions are parallel to the local SQIB [0 0 1] direction.

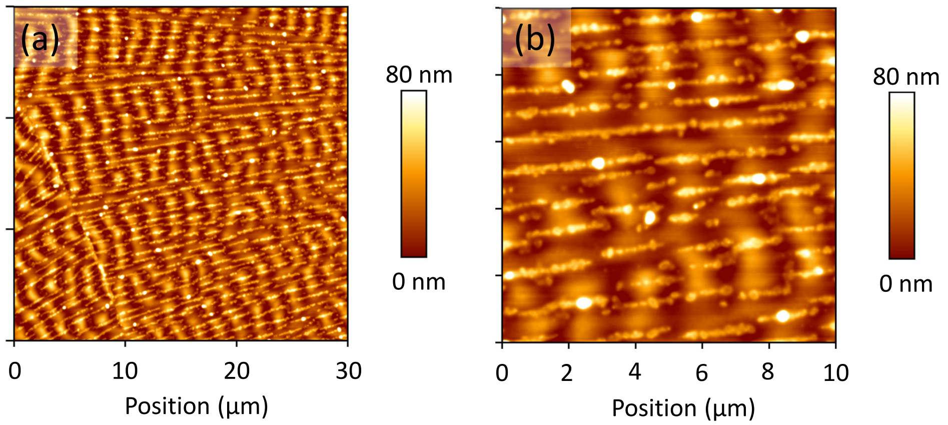

Figure S2: Large-scale (a) and more detailed (b) AFM images of protrusions in the orthorhombic SQIB polymorph.

The average distance and height of the nanodunes depends on the processing parameters, Figure S3. Their morphological dimensions appear to depend on the film thickness; however, the controlling parameters are the acceleration of the spincoater and its final speed, Table S1. The absolute layer thickness can also be controlled by the concentration of the SQIB solution in chloroform, here 6 mg/mL – the higher the concentration, the thicker the layers.

Table S1: Spincoating parameters and resulting layer thicknesses for 6 mg/mL SQIB in chloroform.

Figure S3	spincoating parameters for 60 s	thickness
(a)	1500 rpm reached in 5 s	(50 ± 5) nm
(b)	3000 rpm reached in 3 s	(40 ± 5) nm
(c)	4000 rpm reached in 1 s	(30 ± 4) nm

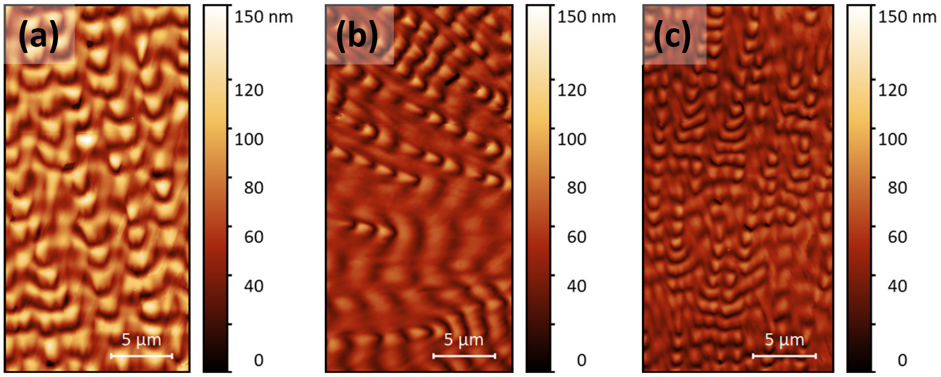

Figure S3: Sample series of SQIB, where the annealing was performed in an inert atmosphere on a preheated hotplate at 180 °C. The decreasing layer thickness was controlled by increasing the spin acceleration and speed from (a) to (c) as listed in Table S1. Nanodune height and periodicity both decrease with increasing spin speed and thus apparently with decreasing film thickness.

Polarized Absorbance

Using the published wavelength-dependent dielectric tensor of the orthorhombic SQIB polymorph,[2] polarization dependent absorbance spectra can be calculated[3] for any contact plane of the thin film. Examples are shown in Figure S4 for the (0 1 0), (0 0 1), and (1 0 0) faces together with the corresponding dielectric functions along the in-plane crystal axes. For polarized absorption spectra of the (1 1 0) and (1 1 1) faces, effective dielectric functions are also shown, Figure S5. Note that rotation of the sample leads to a new dielectric tensor with off-diagonal elements, resulting from mixing of the original diagonal elements for ε. SQIB films show strong absorption in the red. Due to the Kramers-Kronig relations, the peaks in the imaginary part of ε dictate a specific dispersive shape for the real parts across the entire spectrum. When the tensor is rotated, this complex, wavelength- and polarization-dependent behavior is mixed into the off-diagonal elements. The strong absorption features originally confined to the red wavelength region can now influence the optical properties at other wavelengths. Here, it results in a new solution where the imaginary parts of ε are even enhanced at a shorter wavelength. This effect is visible in the calculated absorbance spectra for the (1 1 1) face at $\lambda \approx 530$ nm in Figure S5(b).

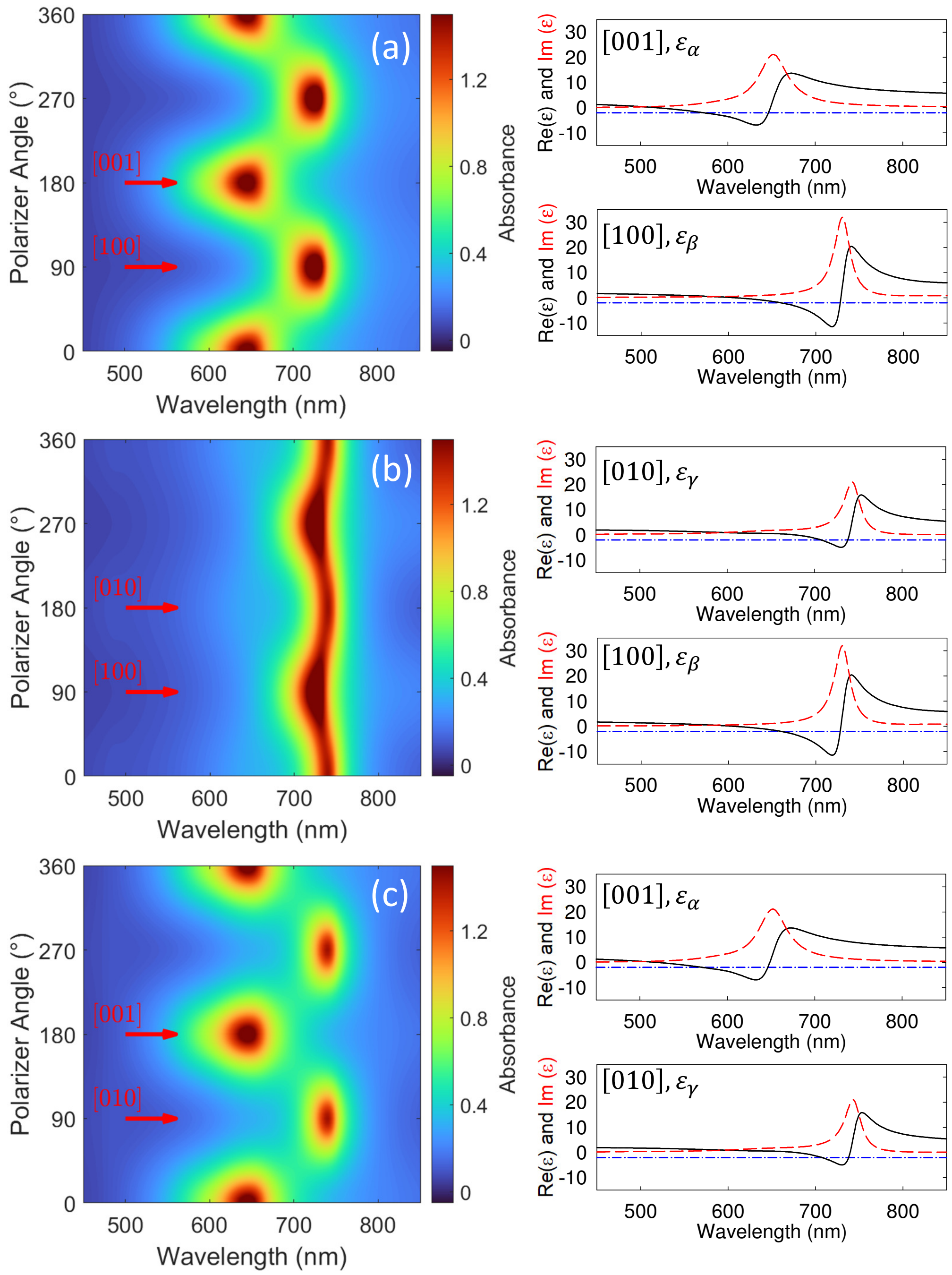

Figure S4: Left column: Calculated[3] normal incidence absorbance spectra for a 50 nm thick film from the orthorhombic SQIB polymorph with its (0 1 0) (a), (0 0 1) (b), and (1 0 0) (c) planes being parallel to the glass substrate (index of refraction $n_{\mathrm{sub}} = 1.52$) and polarization angles between 0° and 360°. On the right panel, the corresponding real and imaginary parts (black solid and red dashed lines, respectively) of the dielectric functions[2] along the in-plane crystal axes are shown. $\mathrm{Re}(\varepsilon) = -2$ is marked by horizontal, dash-dotted blue lines.

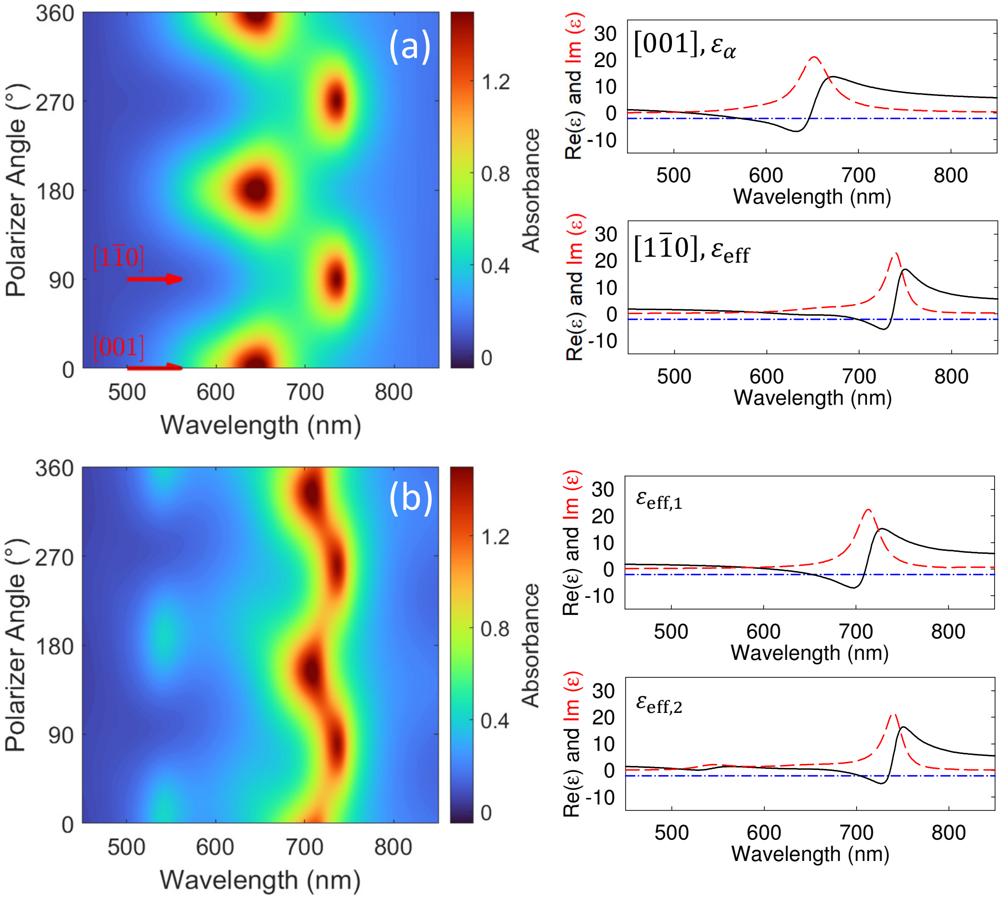

Figure S5: The same as Figure S4, but for (a) the SQIB (1 1 0) plane and (b) the (1 1 1) plane being parallel to the substrate. The calculated absorbance spectrum in (a) is the same as shown in Figure 1(f). $\mathrm{Re}(\varepsilon) = -2$ is marked by a horizontal, dash-dotted blue lines.

Raman Spectroscopy and Raman Mapping

The general Raman tensors R of the A_g, B_{1g}, B_{2g}, and B_{3g} crystal modes for the orthorhombic Pbcn polymorph with D_{2h} crystal point group symmetry are as follows:[4]

$$\begin{aligned}
&R(A_g) = \begin{pmatrix} a_{xx} & 0 & 0 \\ 0 & a_{yy} & 0 \\ 0 & 0 & a_{zz} \end{pmatrix} \\
&R(B_{1g}) = \begin{pmatrix} 0 & a_{xy} & 0 \\ a_{xy} & 0 & 0 \\ 0 & 0 & 0 \end{pmatrix} \quad R(B_{2g}) = \begin{pmatrix} 0 & 0 & a_{xz} \\ 0 & 0 & 0 \\ a_{xz} & 0 & 0 \end{pmatrix} \quad R(B_{3g}) = \begin{pmatrix} 0 & 0 & 0 \\ 0 & 0 & a_{yz} \\ 0 & a_{yz} & 0 \end{pmatrix}
\end{aligned} \tag{S1}$$

The general Raman tensors R of the A_g and B_g crystal modes for the monoclinic P2$_1$/c polymorph (assuming the unique axis is the b-axis) with C_{2h} crystal point group symmetry are as follows:[4]

$$R(A_g) = \begin{pmatrix} a_{xx} & 0 & a_{xz} \\ 0 & a_{yy} & 0 \\ a_{xz} & 0 & a_{zz} \end{pmatrix} \quad R(B_g) = \begin{pmatrix} 0 & a_{xy} & 0 \\ a_{xy} & 0 & a_{yz} \\ 0 & a_{yz} & 0 \end{pmatrix} \tag{S2}$$

The Raman tensors listed above are given for their default orientations. For the respective preferred orientations of the SQIB polymorphs, the tensors can be converted using a Tensor Transformation Tool freely available from the Bilbao Crystallographic Server: `https://www.cryst.ehu.es/cryst/transformtensor.html` (accessed 20.08.2025).

For all Raman spectra shown in the main text, no additional analyzer is part of the detection path. In Figure S6, spectra with horizontal or vertical linear polarizers are exemplarily presented for two selected rotational domains, where (a) the local c -axis is almost vertical (domain 1) and (b) horizontal (domain 2) oriented. The highest Raman intensity is obtained when the excitation polarization is parallel to the local c-axis, where – because of the self-polarization of the grating inside the spectrometer – vertical excitation (domain 1) leads to a stronger signal than horizontal excitation (domain 2).

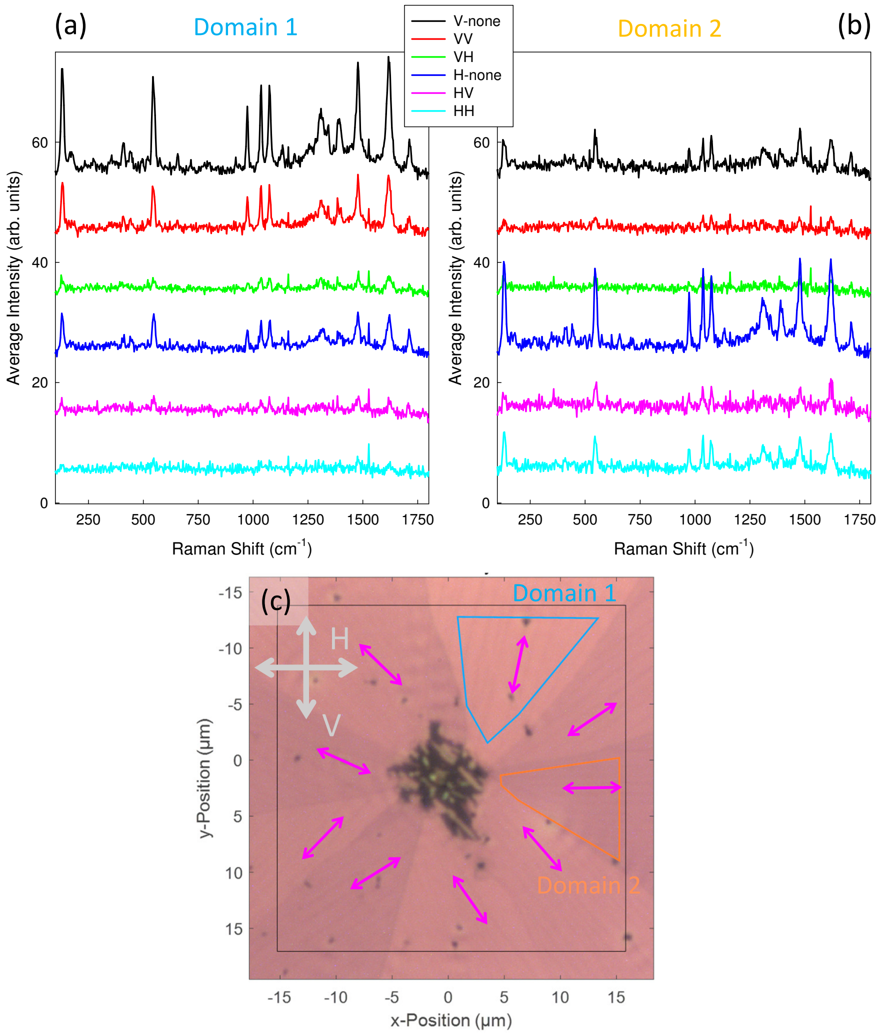

Figure S6: Full polarization resolved sets of spectra from two different SQIB domains from Figure 5. Spectra in (a) are taken from domain 1, spectra in (b) from domain 2. The first letter (H for horizontally, V for vertically) denotes the polarization direction of the excitation, the second letter the polarization of the detection. The pink arrows in the optical image (c) mark the local c-axis, derived from the crack/protrusion directions.

In both domains, a residual signal can also be seen for crossed polarizer/analyzer configurations. For A_g modes, this is possible when they are not aligned with the excitation polarization, since the rotated tensor then obtains values in the off-diagonals. We consider that the Raman signals are dominated by molecular A_g modes (rather than crystalline B_g modes), which have an incoherent angular average along the c-axis but still produce cross-polarized signals, see also Figure S1.

For the monoclinic polymorph, some Raman bands show an additional splitting by 5 cm^{-1} to 10 cm^{-1} compared to the case of the orthorhombic polymorph, Figure S7.

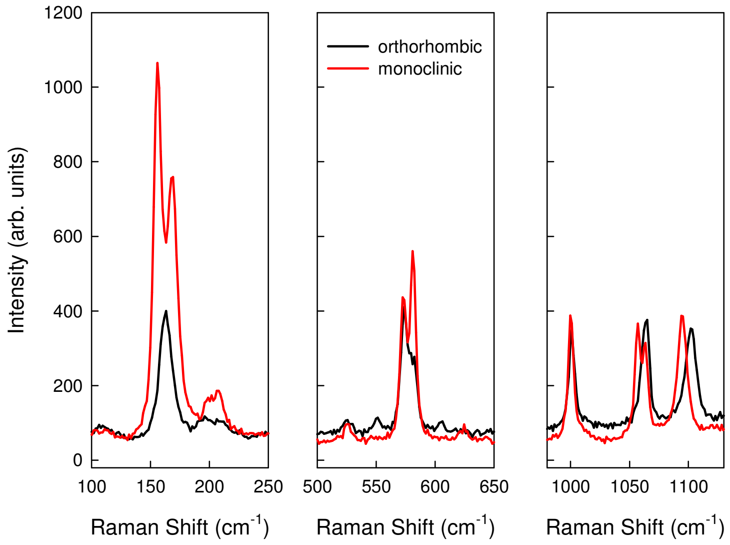

Figure S7: Details of the Raman spectra for the monoclinic and the orthorhombic polymorph, red and black lines, respectively. The bands around 160 cm^{-1} and 1060 cm^{-1} show an additional splitting for the monoclinic case.

Raman mapping demonstrates, that except for the overall intensities, the Raman spectra of the tops and valleys of the nanodunes are identical, Figure S8.

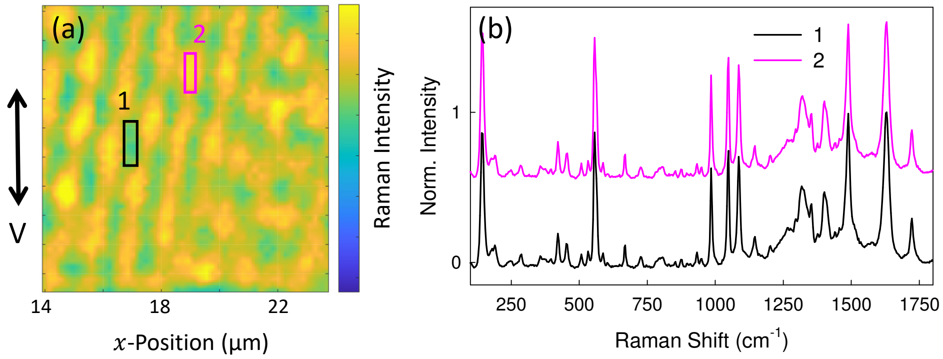

Figure S8: (a) Intensity of the first strong Raman band (120 cm^{-1} to 180 cm^{-1}) of SQIB dunes within a single domain for V-polarized excitation. (b) Averaged Raman spectra for the area in a valley between two dunes (1) and on top of a dune (2). The laser power was 0.5 mW, the magnification of the microscope objective 100×. The pink curve is shifted vertically to ease comparison.

Field Enhancement

The calculation of the electric field enhancement $|E_i/E_0|$ close to a 20 nm radius nanoparticle at the resonance wavelengths for the UDC and LDC, Figures S9(a) and (b) shows the maximum enhancement next to the particle surface. In comparison, illumination of a 20 nm radius Na particle at its dipole resonance leads to an enhancement, which is an order of magnitude larger, Figure S9(c). Note that because of the assumed size of the Na particle, the dipole resonance is already red-shifted by 20 nm compared to the one for a small particle.

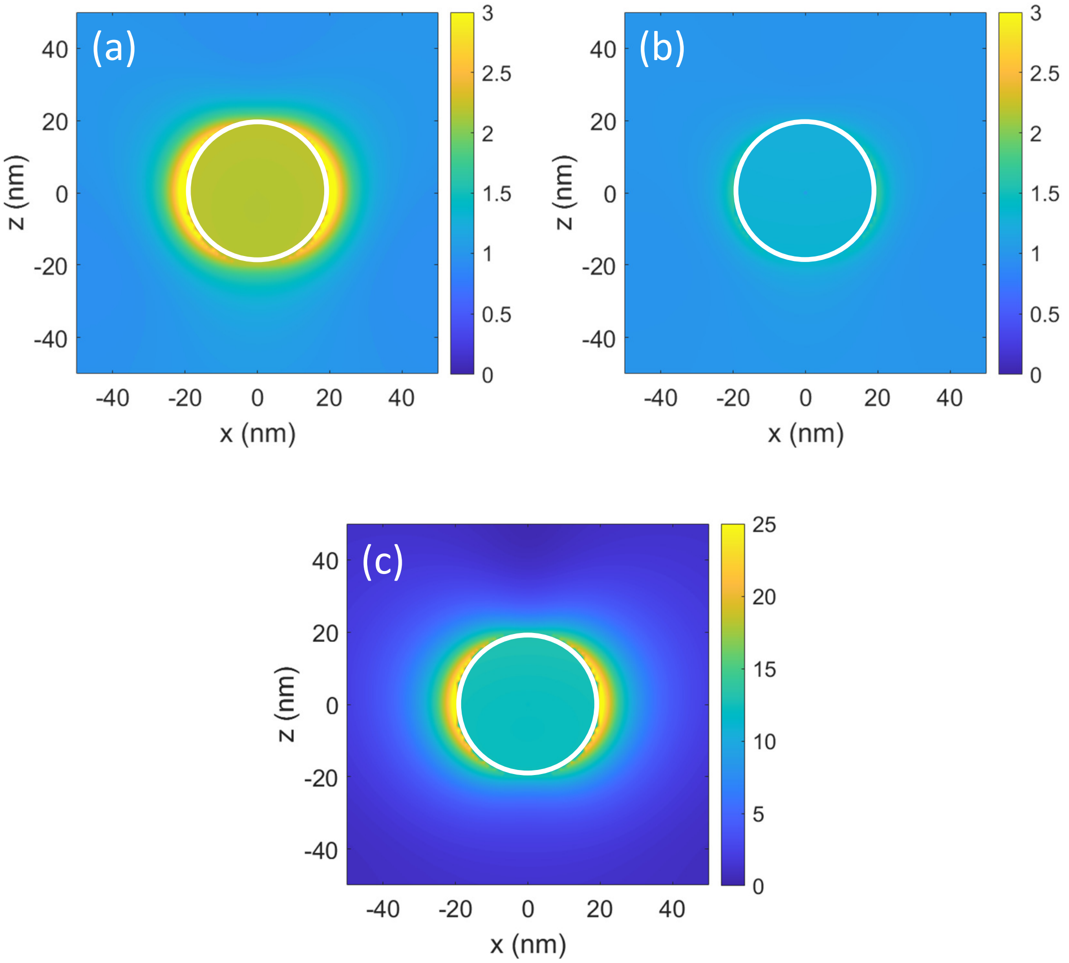

Figure S9: Electric field enhancement $|E_i/E_0|$ in SQIB nanospheres in vacuum, considering either the dielectric function along the [0 0 1] direction (a) or along $[1\,\bar{1}\,0]$ (b).[2,5,6] In (a), the field enhancement for $\lambda = 555$ nm is shown, and (b) for $\lambda = 625$ nm, both for a sphere with 20 nm radius. The direction of the incoming plane wave is along the z-axis. The white circles mark the spherical nanoparticle. As a comparison, the same is plotted in (c) for a spherical Na nanoparticle[7] of 20 nm radius and at its dipole resonance at $\lambda = 398$ nm.

References

(1) Momma, K.; Izumi, F. VESTA 3 for three-dimensional visualization of crystal, volumetric and morphology data. *J. Appl. Crystallogr.* **2011**, *44*, 1272–1276.

(2) Funke, S.; Duwe, M.; Balzer, F.; Thiesen, P. H.; Hingerl, K.; Schiek, M. Determining the Dielectric Tensor of Microtextured Organic Thin Films by Imaging Mueller Matrix Ellipsometry. *J. Phys. Chem. Lett.* **2021**, *12*, 3053–3058.

(3) Bay, M. M.; Vignolini, S.; Vynck, K. PyLlama: A stable and versatile Python toolkit for the electromagnetic modelling of multilayered anisotropic media. *Comput. Phys. Commun.* **2022**, *273*, 108256.

(4) Gregora, I. *International Tables for Crystallography*; International Union of Crystallography, 2013; pp 334–348.

(5) Bohren, C.; Huffman, D. *Absorption and Scattering of Light by Small Particles*; John Wiley & Sons, New York, 1983.

(6) Ma, D.; Tuersun, P.; Cheng, L.; Zheng, Y.; Abulaiti, R. PyMieLab_V1.0: A software for calculating the light scattering and absorption of spherical particles. *Heliyon* **2022**, *8*, e11469.

(7) Smith, N. Optical Constants of Sodium and Potassium from 0.5 to 4.0 eV by Split-Beam Ellipsometry. *Phys. Rev.* **1969**, *183*, 634 – 644.
